# Variations and Interseasonal Changes in the Gut Microbial Communities of Seven Wild Fish Species in a Natural Lake with Limited Water Exchange during the Closed Fishing Season

**DOI:** 10.3390/microorganisms12040800

**Published:** 2024-04-16

**Authors:** Yangyang Liang, Zijia Wang, Na Gao, Xiaoxue Qi, Juntao Zeng, Kai Cui, Wenxuan Lu, Shijie Bai

**Affiliations:** 1 Key Laboratory of Freshwater Aquaculture and Enhancement of Anhui Province, Fisheries Research Institute, Anhui Academy of Agricultural Sciences, Hefei 230001, China; liangyy10214@126.com (Y.L.); gaona9009@sina.com (N.G.); cuikai66@163.com (K.C.); ahfish@126.com (W.L.); 2 Institute of Deep-Sea Science and Engineering, Chinese Academy of Sciences, Sanya 572000, China; wangzj@idsse.ac.cn (Z.W.); qixx@idsse.ac.cn (X.Q.); zengjt@idsse.ac.cn (J.Z.); 3 University of Chinese Academy of Sciences, Beijing 100049, China

**Keywords:** Chaohu Lake, wild fish, gut, microbial communities, seasonal variation, co-occurrence network

## Abstract

The gut microbiota of fish is crucial for their growth, development, nutrient uptake, physiological balance, and disease resistance. Yet our knowledge of these microbial communities in wild fish populations in their natural ecosystems is insufficient. This study systematically examined the gut microbial communities of seven wild fish species in Chaohu Lake, a fishing-restricted area with minimal water turnover, across four seasons. We found significant variations in gut microbial community structures among species. Additionally, we observed significant seasonal and regional variations in the gut microbial communities. The Chaohu Lake fish gut microbial communities were predominantly composed of the phyla Firmicutes, Proteobacteria(Gamma), Proteobacteria(Alpha), Actinobacteriota, and Cyanobacteria. At the genus level, *Aeromonas*, *Cetobacterium*, *Clostridium sensu stricto 1*, *Romboutsia*, and *Pseudomonas* emerged as the most prevalent. A co-occurrence network analysis revealed that *C. auratus*, *C. carpio*, and *C. brachygnathus* possessed more complex and robust gut microbial networks than *H. molitrix*, *C. alburnus*, *C. ectenes taihuensis*, and *A. nobilis*. Certain microbial groups, such as *Clostridium sensu stricto 1*, *Romboutsia*, and *Pseudomonas*, were both dominant and keystone in the fish gut microbial network. Our study offers a new approach for studying the wild fish gut microbiota in natural, controlled environments. It offers an in-depth understanding of gut microbial communities in wild fish living in stable, limited water exchange natural environments.

## 1. Introduction

Fish gut microbiomes play a crucial role in the nutrient metabolism of hosts, such as cholesterol metabolism and trafficking, and also affect the maturation of host epithelial cells as well as the development of mucous-secreting goblet cells and hormone-secreting enteroendocrine cells [1,2]. Moreover, gut microbes are involved in fish immunity and xenobiotic metabolism; for instance, microbes can regulate the production of glycoproteins and diverse vitamins, amino acids, and digestive enzymes in the fish gut. Additionally, microbes can promote the up-regulation of genes related to innate immunity in fish, including serum amyloid A1, C-reactive protein, complement component 3, angiogenin 4, glutathione peroxidase, and myeloperoxidase [1,3,4]. A complex and integrated interaction between the epithelium, immune components in the mucosa, and microbes is responsible for the development and maturation of the gut-associated immune system of the host [4,5]. The intestinal tract of fish, as well as the skin and gills, are major pathways for several pathogens to enter and form fatal infections, and the intestinal tract of fish is thought to be the primary route for the development of diseases such as vibriosis, furunculosis, enteric septicemia, and aeromoniasis in fish [4,6]. The gut of fish typically contains a wide range of bacteria with pathogen-suppressive capabilities, and the gut can protect the host by depriving invading pathogens of nutrients and secreting a range of antimicrobial substances [4,7].

Although previous studies have shown that the fish microbiota is involved in a number of important biological functions such as physiological, nutritional and immune processes [4], there are at least 28,000 species of fish in the world, representing almost half of all extant vertebrates [8], so revealing and understanding the community structure and composition of the gut microbes of these fishes is a daunting task, with the main difficulty being the acquisition of representative healthy samples. The silver carp (*Hypophthalmichthys molitrix*) is one of the four major domesticated fish in China [9], and it is a filter-feeding fish that feeds mainly on phytoplankton. Previous studies have found that *Cetobacterium* and *Aeromonas* are the major microbial taxa in the gut of silver carp, as well as a large number of unclassified microbial linages [10]. The bighead carp (*Aristichthys nobilis*) is also a filter-feeding fish native to China and is widely distributed, and has even been used to control outbreaks of blooms in freshwater due to its filter feeding of planktonic algae [11]. Furthermore, *Dielma*, *Cetobacterium*, *Aeromonas*, *Clostridium XI*, and some unclassified microbial taxa were found to be the main groups of gut microorganisms in bighead carp, which feeds exclusively on aquatic phytoplankton in its natural state [10,12,13]. The crucian carp (*Carassius auratus*) is an omnivorous fish that is widely cultured in Chinese freshwaters and is often used as a model organism for toxicological studies because of its suitable size, ease of reproduction, and ecological relevance [14,15]. The intestinal tract of healthy crucian carp contained more *Cetobacterium*, *GpXIII*, *Steroidobacter*, *Clostridium XI*, *GpIIa*, *Gp17*, *Clostridium sensu stricto*, and some unclassified cyanobacteria than those of diseased crucian carp [16]. The common carp (*Cyprinus carpio*) has been farmed in China for more than 2500 years, and common carp is another common freshwater omnivorous fish, but its diet is more carnivorous, feeding mainly on benthic organisms [17], and the intestinal microorganisms of the common carp are mainly composed of *Cetobacterium*, *Aeromonas*, *Clostridium XI*, and *Clostridium sensu stricto* [10]. In addition, there are predatory fish such as the carnivorous topmouth culter (*Culter alburnus*), *coilia brachygnathus*, and lake anchovy (*Coilia ectenes taihuensis*), all of which prey on small fish and shrimp in their environment. Limited studies have shown that the gut microbial community of the topmouth culter is mainly composed of *Cetobacterium* and *Aeromonas* [18]. *Clostridium sensu stricto 1* is the dominant microbial taxon in the gut of *coilia brachygnathus* [19]; however, the gut microbial community of the lake anchovy at the genus level consists predominantly of *Halomonas*, *Pseudomonas*, *Clostridium sensu stricto 1*, and *Ochrobactrum* [20].

Although gut microbes play a crucial role in fish nutrition and metabolism, their composition and abundance are strongly influenced by many direct and indirect factors. Many studies have shown that gut microbial communities vary according to host individual development [21], diet [12,22], environment [19,23], and host genetics and phylogeny, which refer to fish populations from different geographic clusters having different gut microbiomes and fish genotypes correlating with gut microbiomes, with the more genetically distinct populations exhibiting greater differences in gut microbiomes [24,25]. However, previous studies have focused on single species in limited environments, such as fish in farmed ponds or under laboratory conditions and fed specific foods [12,15,18,26]. Other studies have focused on single or multiple species in natural environments, but either in more open waters with unstable aquatic conditions or single fish at a single point in time in inland lakes where water exchange is not intense [19,20]. The samples collected in this way cannot simultaneously satisfy the diversity and composition of the gut microbial communities of different wild fish in different seasons under the same relatively stable living environment and similar food sources of fish. Therefore, we can reveal the gut microbial communities of these wild fish in a more systematic and comprehensive way based on larger-scale samples of different wild fish collected in multiple sampling periods under relatively constant environmental conditions, such as inland lakes with little water exchange, and at the same time, we can carry out more meaningful and accurate ecological comparisons of gut microbiology of different wild fish.

Chaohu Lake is located on the north shore of the lower reaches of the Yangtze River and is one of the five largest freshwater lakes in China. The watershed area of Chaohu Lake is about 13,500 km^2^, and it used to be naturally connected to the Yangtze River. However, 63 years ago and 57 years ago, the Chaohu Lock and Yuxi Lock were built, respectively, blocking the natural connection between Chaohu Lake and the Yangtze River, and thus Chaohu Lake became a semi-enclosed lake with an artificially controlled water level. According to the characteristics of Chaohu Lake, which has less water exchange and a more stable environment, we collected a large number of samples from seven different wild fishes in Chaohu Lake during the spring, summer, autumn, and winter seasons to address five scientific questions: 1. whether the gut microbial community structure of different wild fish varies with seasonal changes in a relatively closed water and living environment, 2. the characteristics of the gut microbial community composition of these seven wild fishes in Chaohu Lake, 3. whether the structural characteristics of the gut microbial communities of different fish species show similar patterns with the similarities and differences in food habits, 4. whether there are significant differences between the microbial communities of the foregut and hindgut in fish guts, and 5. what the structural characteristics are of the gut microbial community network of these seven wild fish species in Chaohu Lake.

## 2. Materials and Methods

### 2.1. Sample Collection

**Figure 1:**
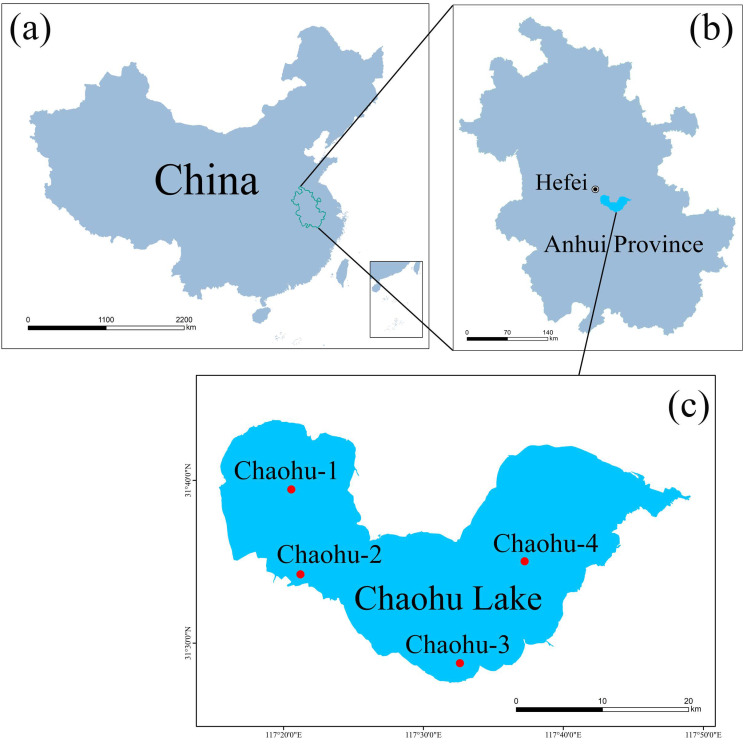
A map of the Chaohu Lake and the sampling sites in this study. Chaohu Lake is located in Hefei, Anhui Province, China. We collected intestinal tracts of seven different wild fish species during four consecutive seasons in Chaohu Lake. (**a**) The location of Anhui Province on the map of China; (**b**) The location of Hefei City on the map of Anhui Province; (**c**) The shape of Chaohu Lake and the location of the four samplings in Chaohu Lake.

We collected samples of seven species of wild fish, silver carp (*Hypophthalmichthys molitrix*), bighead carp (*Aristichthys nobilis*), crucian carp (*Carassius auratus*), topmouth culter (*Culter alburnus*), *coilia brachygnathus*, common carp (*Cyprinus carpio*), and lake anchovy (*Coilia ectenes taihuensis*), living in Chaohu Lake on 30 October 2019, 20 December 2019, 27 March 2020, and 30 June 2020, representing a consecutive autumn, winter, spring, and summer season, respectively (Figure 1). Meanwhile, the foregut and hindgut samples of these fishes were collected using sterile scissors and forceps, but due to the short intestinal tract of the lake anchovy, it was not possible to distinguish the foregut and hindgut well, so we took the whole intestinal tract of each lake anchovy as an independent sample, and the specific sampling information is shown in Table 1.

### 2.2. DNA Extraction and Sequencing

The gut microbial DNA of fish was extracted with a DNeasy PowerSoil Pro Kit (QIAGEN, Germantown, MD, USA) according to the manufacturer’s instructions. The extracted DNA was quantified using a Qubit fluorometer (Invitrogen Inc. Manufacturer: Life Technologies Holdings Pte Ltd., Singapore), and the hypervariable regions V3–V4 of the 16S rRNA gene were amplified using the primer pair 338F (5′-ACTCCTACGGGAGGCAGCAG-3′) and 806R (5′-GGACTACHVGGGTWTCTAAT-3′). The PCR cycling conditions were as follows: denaturation at 95 °C for 3 min, followed by 27 cycles at 95 °C for 30 s, 55 °C for 30 s, 72 °C for 45 s, and a final extension at 72 °C for 10 min. Products of the triplicate PCR reactions were combined after purification using the TaKaRa purification kit (TaKaRa, Shiga, Japan). The PCR products were prepared for library construction using the TruSeq DNA sample preparation kit (Illumina, San Diego, CA, USA) according to the manufacturer’s instructions. These libraries were sequenced at MajorBio Co. Ltd. (Shanghai, China) using the HiSeq platform (Illumina) with paired-end 300 bp sequence reads. Raw sequencing reads for all samples were deposited in the NCBI database (http://www.ncbi.nlm.nih.gov/ accessed on 21 March 2024) under BioProject accession number PRJNA1061160 for the microbial datasets of all fish guts in this study.

### 2.3. Microbial Data Processing and Statistical Analysis

After completing the sequencing and obtaining the raw data, sequences were sorted to individual samples according to barcodes, allowing for one mismatch, after which the barcodes as well as forward and reverse primer sequences were removed from the sequences to obtain clean data. We used FLASH (version 1.2.8) [27] to obtain paired-end full-length sequences of sufficient length, with at least 30 bp of overlap. We then used Btrim (version 0.2.0) to select high-quality sequences without Ns and between 400 bp and 435 bp in length for subsequent analyses [28]. UNOISE3 was used to generate amplicon sequence variants (ASVs) with default settings [29] without singletons, and subsequently a representative sequence from each ASV was selected for taxonomic annotation, and taxonomic information was obtained using the RDP classifier for comparison with the SILVA 138 database including bacterial, archaeal, and eukaryotic sequences [30]. The generated ASV table was used in the subsequent analyses. The diversity of microbial communities in different parrotfish gut samples was determined by a statistical analysis of α-diversity indices. Shannon’s and inverse Simpson’s indices were calculated using the vegan package in R language version 4.3 [31]. Chao1 values [32] were generated using the Mothur program [33]. A molecular ecological network analysis (MENA) was used to perform the structure of microbial community networks [34,35]. Only the ASVs that appeared in more than half of the fish gut samples of each group were included in the network analysis. Correlations were calculated using the Spearman coefficient, and a random matrix theory (RMT)-based approach was employed to delimit the microbial network interactions between samples. The keystone taxa were allocated according to the within-module connectivity (Zi) and among-module connectivity (Pi) according to a previously used method [34]. Nodes (ASVs) can be divided into four categories: (1) peripherals, which includes the nodes with Zi ≤ 2.5 and Pi ≤ 0.62, indicating nodes interconnected by a few links within the modules; (2) connectors, which includes the nodes with Zi ≤ 2.5 and Pi > 0.62, indicating nodes linking to various modules; (3) module hubs, which includes the nodes with Zi > 2.5 and Pi ≤ 0.62, indicating nodes within the modules are highly connected; and (4) network hubs, which includes the nodes with Zi > 2.5 and Pi > 0.62, indicating nodes highly connected among modules. To compare the similarities and differences in the gut microbial community structure of these seven wild fish species in Chaohu Lake, grouping comparisons based on species differences, seasonal differences, and differences in gut sites, we used the non-metric multidimensional scaling (NMDS) method, a statistical tool based on ß-diversity, to calculate the Bray–Curtis and Jaccard distance matrices. We also tested whether there were any significant dissimilarities in the gut microbial community structure of these fish by performing a permutational multivariate analysis of variance (PERMANOVA) and multi-response permutation procedure (MRPP) on the gut microbial community structure of these fish in different groupings. Data comparisons between different groups were performed by the Mann–Whitney U test of IBM SPSS Statistics 19.

## 3. Results

### 3.1. Sequencing Statistics and Microbial Diversity

**Figure 2:**
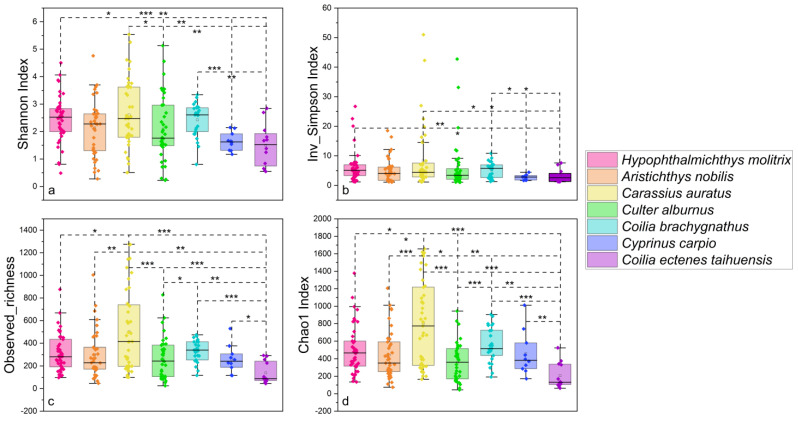
Comparisons of four α-diversity indices. We calculated and compared these four α-diversity indices for the 210 gut microbial communities of seven wild fish species from Chaohu Lake: (**a**) Shannon’s index, (**b**) inverse Simpson’s index, (**c**) observed abundance, and (**d**) Chao1 index. The significant differences were tested by the Mann–Whitney U test: (*) *p* < 0.05, (**) *p* < 0.01, (***) *p* < 0.001.

**Figure 3:**
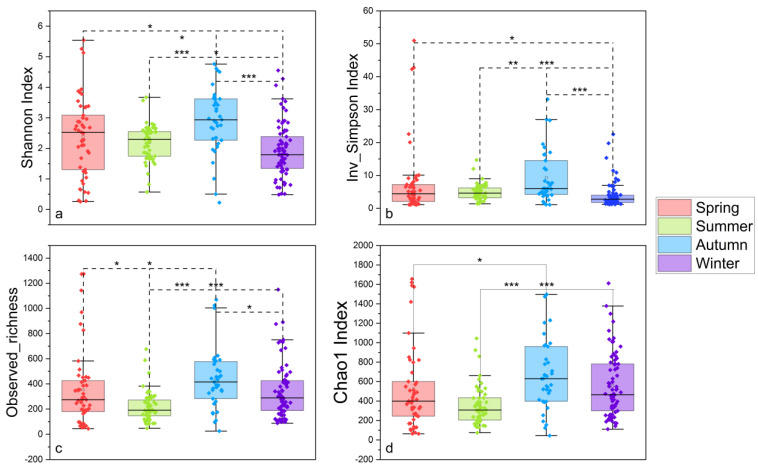
Comparisons of four α-diversity indices. We calculated and compared these four α-diversity indices for the 210 gut microbial communities of seven wild fish species from Chaohu Lake by season: (**a**) Shannon’s index, (**b**) inverse Simpson’s index, (**c**) observed abundance, and (**d**) Chao1 index. The significant differences were tested by the Mann–Whitney U test: (*) *p* < 0.05, (**) *p* < 0.01, (***) *p* < 0.001.

**Figure 4:**
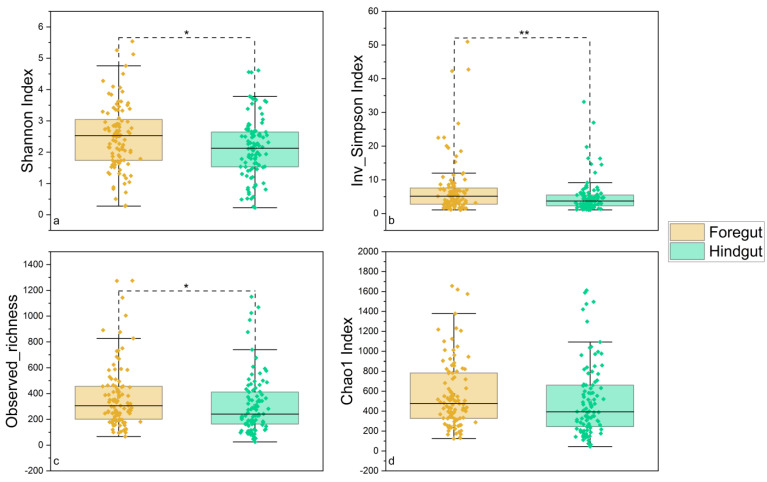
Comparisons of four α-diversity indices. We calculated and compared 210 gut microbial communities of seven wild fish species from Chaohu Lake by different parts of the gut: (**a**) Shannon’s index, (**b**) inverse Simpson’s index, (**c**) observed abundance, and (**d**) Chao1 index. The significant differences were tested by the Mann–Whitney U test: (*) *p* < 0.05, (**) *p* < 0.01.

After quality control, a total of 7,833,191 sequences were obtained from 210 gut samples of seven wild fish species from Chaohu Lake. In order to obtain more accurate α-diversity results to analyze microbial diversity, composition, and structure, we refined each sample to 17,248 sequences and then calculated the α-diversity of the gut microbial community of these fish. The results showed that *C. ectenes taihuensis* had the lowest α-diversity of gut microbial communities, followed closely by *C. carpio*, which also possessed low α-diversity, and conversely the fish with higher α-diversity of gut microbial communities were *H. molitrix*, *C. auratus*, and *C. brachygnathus* (Figure 2). Grouped by season, the diversity of gut microbial communities in fish from Lake Chaohu was higher in autumn and winter than in summer and spring. The highest diversity of gut microbes was found in autumn, while the lowest diversity of gut microbes was found in summer (Figure 3). In addition, overall, the diversity of microbial communities in the foregut of fish in Chaohu Lake was higher than that in the hindgut (Figure 4).

### 3.2. Gut Microbial Community Composition of Seven Wild Fish Species from Chaohu Lake

**Figure 5:**
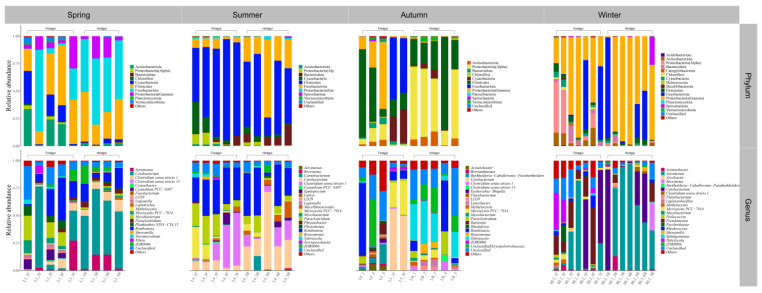
The composition of microbial communities in the foregut and hindgut of *H. molitrix* in Chaohu Lake in spring, summer, autumn, and winter at the classification level of phylum and genus.

**Figure 6:**
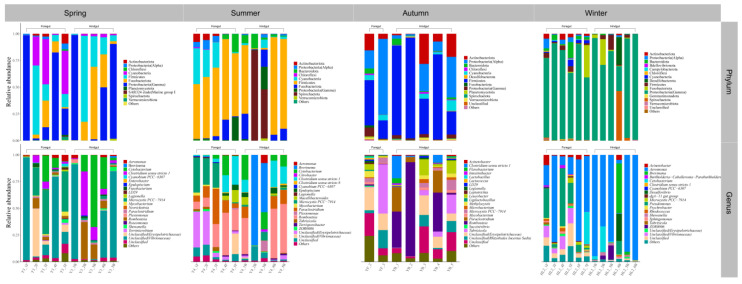
The composition of microbial communities in the foregut and hindgut of *A. nobilis* in Chaohu Lake in spring, summer, autumn, and winter at the classification level of phylum and genus.

**Figure 7:**
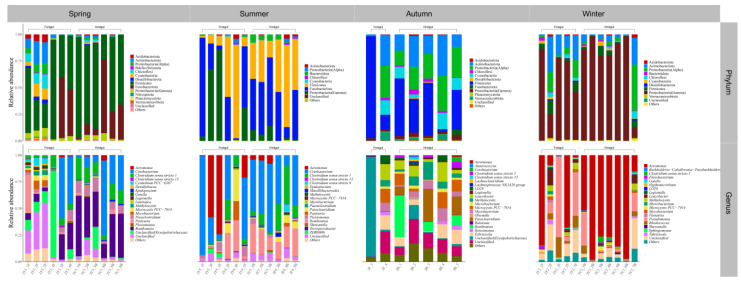
The composition of microbial communities in the foregut and hindgut of *C. auratus* in Chaohu Lake in spring, summer, autumn, and winter at the classification level of phylum and genus.

**Figure 8:**
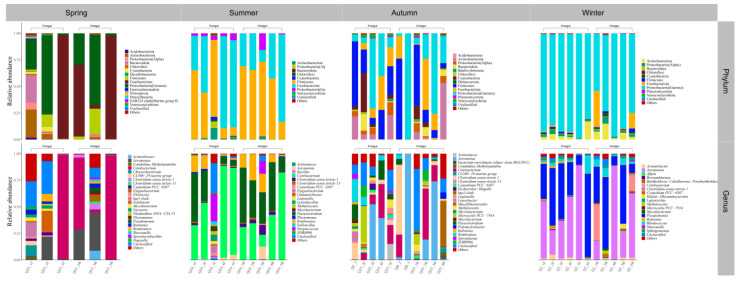
The composition of microbial communities in the foregut and hindgut of *C. alburnus* in Chaohu Lake in spring, summer, autumn, and winter at the classification level of phylum and genus.

**Figure 9:**
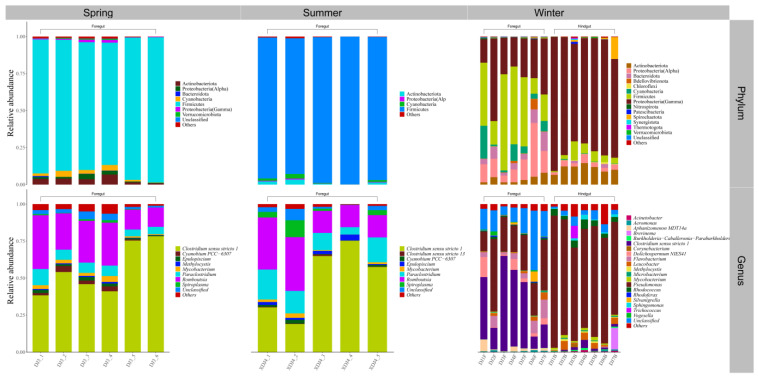
The composition of microbial communities in the foregut and hindgut of *C. brachygnathus* in Chaohu Lake in winter and foregut in spring and summer at the classification level of phylum and genus.

**Figure 10:**
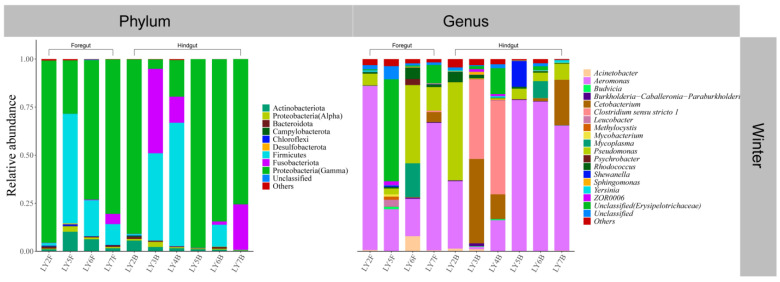
The composition of microbial communities in the foregut and hindgut of *C. auratus* in Chaohu Lake in winter at the classification level of phylum and genus.

**Figure 11:**
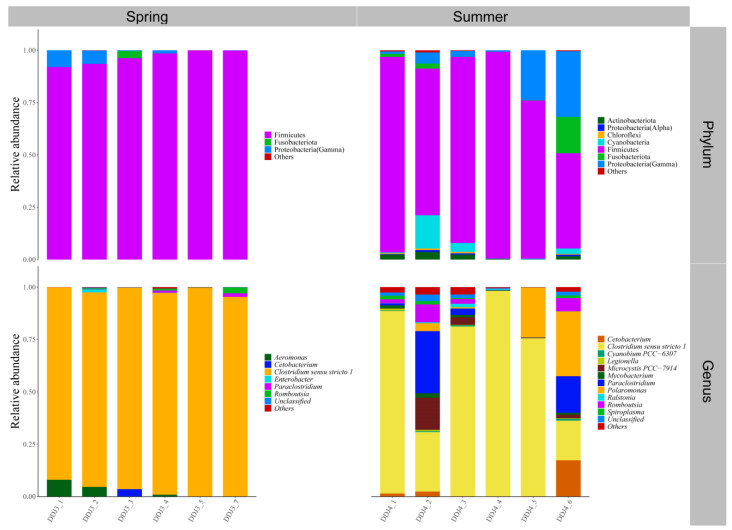
The composition of microbial communities in the hindgut of *C. ectenes taihuensis* in Chaohu Lake in spring and summer at the classification level of phylum and genus.

The relative abundance of gut microbes was apparent at the phylum and genus levels, with a similarity of 97% for ASV taxonomy, and provided detailed relative abundance information on gut microbial community composition (Figure 5, Figure 6, Figure 7, Figure 8, Figure 9, Figure 10 and Figure 11). In the spring, the foregut of *H. molitrix* was dominated by Firmicutes, Fusobacteriota, Actinobacteriota, and Cyanobacteria at the taxonomic level of phylum, whereas the hindgut was predominantly dominated by Fusobacteriota, Firmicutes, and Proteobacteria(Gamma). Additionally, at the taxonomic level of genus, the main dominant taxa of its foregut microbial community were *Cetobacterium*, *Romboutsia*, *Mycobacterium*, and *Aeromonas*, whereas the hindgut was predominantly dominated by *Cetobacterium*, *Romboutsia*, and *Aeromonas* (Figure 5). In the summer, the main taxa in the foregut of chub at the taxonomic level of phylum became Firmicutes, Cyanobacteria, and Fusobacteriota, while in the hindgut mainly Firmicutes, Fusobacteriota, and Bacteroidota dominated. At the taxonomic level of genus, the microbial community in the foregut of chub in summer consisted mainly of *Romboutsia*, *Clostridium sensu stricto 1*, *Paraclostridium*, and *Microcystis PCC-7914*, while in the hindgut the dominant taxa were mainly *Cetobacterium*, *Romboutsia*, *Clostridium sensu stricto 1*, and *Paraclostridium* (Figure 8). In the fall, the dominant taxa in the foregut of *H. molitrix* at the taxonomic level of phylum were Firmicutes, Fusobacteriota, and Proteobacteria(Gamma), while the hindgut was dominated by Proteobacteria(Alpha) and Firmicutes. At the taxonomic level of genus, the main dominant taxa in the microbial community in the foregut of chub in the fall were *Cetobacterium*, *Romboutsia*, and *Microcystis PCC-7914*, while in the hindgut they were dominated by *Tabrizicola* and unclassified lineages from *Erysipelotrichaceae*, of which a certain percentage of microbial taxa also occupied the foregut (Figure 5). In the winter, at the taxonomic level of phylum, the predominant taxa in the foregut of chub were composed of Proteobacteria(Gamma), Fusobacteriota, and Proteobacteria(Alpha), whereas the hindgut was dominated by Proteobacteria(Gamma) and Fusobacteriota. At the taxonomic level of genus, the main dominant taxa of the microbial community in the foregut of chub in winter were *Aeromonas*, *Cetobacterium*, *Pseudomonas*, and *Tabrizicola*, whereas the dominant microbial taxa in the hindgut were predominantly *Aeromonas* and *Cetobacterium* (Figure 5).

The composition of the foregut microbial community of *A. nobilis* in Chaohu Lake varied with the seasons as follows: Proteobacteria(Gamma), Cyanobacteria, and Firmicutes dominated the foregut in the spring; Proteobacteria(Gamma) gradually decreased in the summer, while Cyanobacteria and Firmicutes became the major microbial groups in the foregut; Proteobacteria(Alpha) and Firmicutes dominated the foregut microbial communities in the fall; and Proteobacteria(Gamma) became the dominant microbial taxa in the foregut of *A. nobilis* in the winter. At the taxonomic level of genus, the dominant microbial taxa in the foregut of *A. nobilis* in spring were some unclassified lineages from *Vibrionaceae* and *Erysipelotrichaceae* and *Microcystis PCC-7914*. In the summer, the microbial community of the foregut of *A. nobilis* was mainly composed of *Romboutsia*, *Microcystis PCC-7914*, *Paraclostridium*, and unclassified taxa from *Erysipelotrichaceae*, and the foreguts of *A. nobilis* were dominated by *Tabrizicola* and unclassified microorganisms from *Erysipelotrichaceae* in the fall, which changed to a foregut microbial community dominated by *Aeromonas* and *Pseudomonas* as winter progressed. In the hindgut of the *A. nobilis*, the dominant microbial group in the spring was replaced by Fusobacteriota in the phylum Cyanobacteria, and at the genus level it consisted of unclassified taxa from the family *Vibrionaceae* as well as *Cetobacterium*. In the summer, Spirochaetota in the hindgut became the dominant microbial group in the microbial community, and at the genus level it consisted of *Brevinema* and *Romboutsia*. In the fall, the dominant microorganisms in the hindgut of the *A. nobilis* were dominated by *Romboutsia*, and in the winter, *Aeromonas* dominated the microbial community in the hindgut of the *A. nobilis* (Figure 6).

The dominant bacterial groups in both the foregut and hindgut of *C. auratus* in Chaohu Lake in spring belonged to Firmicutes and Fusobacteriota. At the genus level of classification, the microbial community in the foregut of *C. auratus* mainly consisted of unclassified lineages from the family of *Erysipelotrichaceae* and *Cetobacterium*, whereas the main dominant microorganisms in the hindgut were *Romboutsia* and *Cetobacterium*. When entering the summer season, the dominant bacterial taxa in the microbial community in its foregut were Fusobacteriota, Firmicutes, and Proteobacteria(Gamma) at the taxonomic level of phylum, and in its hindgut, they were Fusobacteriota and Firmicutes; moreover, at the level of genus, the foregut was mainly composed of *Cetobacterium* and *Aeromonas*, and the hindgut was dominated by *Cetobacterium* and *Romboutsia*. In the fall, the dominant microorganisms in both the foregut and hindgut of *C. auratus* were Firmicutes, Proteobacteria(Alpha), and Actinobacteriota. At the taxonomic level of genus, the dominant bacterial communities of the hindgut were *Romboutsia*, *Paraclostridium*, and *Leucobacter*, while the foregut was mainly dominated by unclassified taxa from the family *Erysipelotrichaceae*, and into the winter, Proteobacteria(Gamma) was the dominant lineage in the microbial community of foregut and hindgut of the *C. auratus*; the difference was that *Aeromonas* and Pseudomonas were the dominant groups in the foregut, while *Aeromonas* was the absolute dominant taxon in the hindgut (Figure 7).

Both the foregut and hindgut of *C. alburnus* in Chaohu Lake were dominated by Proteobacteria(Gamma) and Fusobacteriota as the main bacterial taxa in the spring, and *Cetobacterium* and *Aeromonas* were the dominant microbial taxa at the genus level; in the summer, at the level of phylum classification, *C. alburnus* was dominated by Fusobacteriota and Firmicutes, which were reflected at the genus level as *Cetobacterium* and *Paraclostridium*. In the fall, the gut microbial communities of *C. alburnus* were dominated by Proteobacteria (Gamma) and Firmicute, and in winter, they were dominated by *Aeromonas* and *Pseudomonas*, which were both affiliated with Proteobacteria(Gamma) as the dominant microbial taxon (Figure 8). The gut microbial community composition of *C. brachygnathus* in Chaohu Lake was dominated by Firmicutes in both spring and summer, and at the genus level was mainly composed of *Clostridium sensu stricto 1* and *Romboutsia*. In winter, the foregut of the *C. brachygnathus* consisted mainly of *Clostridium sensu stricto 1* and *Pseudomonas*, which were affiliated with Firmicutes and Proteobacteria(Gamma), respectively, while in the hindgut *Pseudomonas* was absolutely dominant (Figure 9). Additionally, at Chaohu Lake in winter, the microbial community composition of the foregut and hindgut of *C. carpio* showed differences, consisting mainly of Proteobacteria (Gamma), Firmicutes, and Fusobacteriota in the hindgut and *Aeromonas*, *Clostridium sensu stricto 1*, and *Cetobacterium* at the genus level, whereas the foregut consisted mainly of *Aeromonas* and *Pseudomonas*, which were affiliated with Proteobacteria (Gamma), and some unclassified linages from *Erysipelotrichaceae* (Figure 10). Moreover, the gut microbial communities of *C. ectenes taihuensis* in Lake Chaohu were dominated by *Clostridium sensu stricto 1*, which was affiliated with Firmicutes, both in spring and summer (Figure 11).

### 3.3. Gut Microbial Community Structure of Seven Wild Fish Species from Chaohu Lake

**Figure 12:**
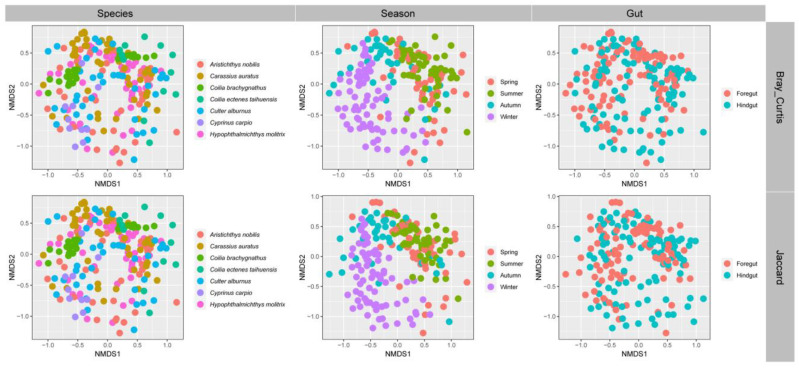
NMDS analysis of gut microbial communities grouped by species, different seasons, and different intestinal parts. Results are based on the ASVs datasets, and upper and lower plots were calculated based on Bray–Curtis dissimilarity index and Jaccard similarity index, respectively.

**Figure 13:**
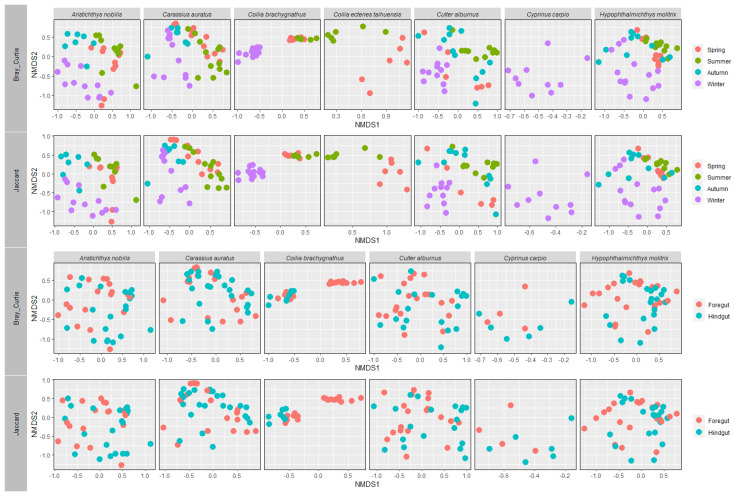
An NMDS analysis of the gut microbial communities grouped by different seasons and different intestinal parts was performed for seven different fish species in Chaohu Lake. The results were calculated based on the ASVs datasets and the Bray–Curtis dissimilarity index and Jaccard similarity index, respectively.

We grouped the gut microbial communities of seven wild fish species in Chaohu Lake according to species, different seasons, and different intestinal parts, respectively. The NMDS analysis and statistical analysis showed that there were significant differences in the gut microbial community structures of these seven different fish species, and not only that, but also, if grouped according to the seasons, the gut microbial community structures of all the fish species in different seasons showed significant differences. Also, overall, the microbial community structures in the foregut and hindgut of these fish were significantly different (Figure 12). In addition, we also analyzed the microbial community structures of different fish individually. The results showed that there was a significant difference in the microbial community structure of the foregut and hindgut in the *H. molitrix*, *A. nobilis*, *C. auratus*, *C. alburnus*, and *C. carpio*, but these five species of fish showed a non-significantly different microbial community structure of the gut in different seasons, and there was no significant difference in the structure of the microbial communities of the gut of the remaining two species of fish in either the foregut and hindgut or in different seasons (Figure 13).

### 3.4. Co-Occurrence Network Profile of Gut Microbial Communities

**Figure 14:**
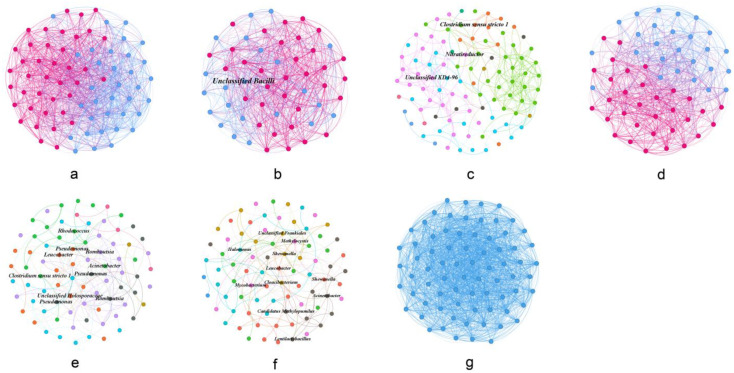
Co-occurrence network of gut microbial communities of seven different fish species in Chaohu Lake. (**a**) *H. molitrix*, (**b**) *A. nobilis*, (**c**) *C. auratus*, (**d**) *C. alburnus*, (**e**) *C. brachygnathus*, (**f**) *C. carpio*, (**g**) *C. ectenes taihuensis*. Different colors indicate different modules. Identified keystone taxa are annotated at the genus level.

We used the MENA method to construct the network structure of the gut microbial communities of the seven fish species in Chaohu Lake to reveal the correlation between the gut microbes of each fish species and also to test the complexity and stability of the gut microbial communities. The co-occurrence network diagrams of the gut microbial communities of seven different fish species in Chaohu Lake and the related parameters, including the numbers of nodes and links, the average clustering coefficient (avgCC), the average path distance (GD), and the modularity (M), are provided (Table 2 and Figure 14). These results showed that the complexity and stability of the gut microbial community networks of these seven fish species in Chaohu Lake were ranked from high to low in the order of *C. auratus*, *C. carpio*, *C. brachygnathus*, *C. alburnus*, *H. molitrix*, *A. nobilis*, and *C. ectenes taihuensis*. Additionally, connectors (Zi ≤ 2.5, Pi > 0.62) and module hubs (Zi > 2.5, Pi ≤ 0.62) in the co-occurrence network of each gut microbial community were selected as the keystone taxa. The results showed that the unclassified lineage of the class Bacilli was the keystone taxon (module hub) in the gut microbial co-occurrence network of *A. nobilis* (Figure 14b). In the gut microbial network of *C. auratus*, the ASVs of *Clostridium sensu stricto 1* and *Nitratireductor* were the keystone taxa as the connectors, and the unclassified KD4-96, from the phylum Chloroflexi, was another keystone taxon as the module hub. The connectors and module hubs also both existed in the gut microbial co-occurrence network of *C. carpio* in Chaohu Lake, which included *Candidatus Methylopumilus*, *Methylocystis*, *Lentilactobacillus*, *Acinetobacter*, *Shewanella*, *Cloacibacterium*, *Halomonas*, *Leucobacter*, and *Mycobacterium*. The keystone taxa of *C. brachygnathus* only included connectors, including the lineages of *Leucobacter*, *Acinetobacter*, *Pseudomonas*, *Romboutsia*, *Rhodococcus*, *Clostridium sensu stricto 1*, and *Holosporaceae* (Figure 14e). However, no keystone taxon was found in the gut microbial co-occurrence network of *H. molitrix*, *C. alburnus*, or *C. ectenes taihuensis* (Figure 14). Moreover, two, two, seven, two, seven, seven, and 1 module were found in the gut microbial co-occurrence network of *H. molitrix*, *A. nobilis*, *C. auratus*, *C. alburnus*, *C. brachygnathus*, *C. carpio*, and *C. ectenes taihuensis*, respectively. The gut microbial co-occurrence networks of *H. molitrix*, *A. nobilis*, and *C. alburnus* showed that they all formed two modules, which were essentially modular microbial communities dominated by *Aeromonas* or *Cetobacterium*, respectively, while *Microcystis PCC-7914*, *Romboutsia*, and *Paraclostridium* also occupied a relative proportion in different modules, respectively (Figures S1, S2 and S4). In the gut microbial network of *C. auratus*, *Clostridium sensu stricto 1*, *Romboutsia*, *Cetobacterium*, and *Aeromonas* dominated the different microbial module communities, respectively, while the rest of the modules consisted mainly of unclassifiable microbial taxa (Figure S3). In addition, unlike the composition of the gut microbial co-occurrence network module of *C. auratus*, in the gut microbial co-occurrence network module of *C. brachygnathus*, in spite of having the same module as *Clostridium sensu stricto 1* and some unclassifiable microbes as the dominant taxa, *Pseudomonas*, *Leucobacter*, and *Mycobacterium* were also distributed as dominant microbial taxa in different microbial modular communities (Figure S5). However, only one module existed in the co-occurrence network of *C. ectenes taihuensis*, and *Clostridium sensu stricto 1* was the main microbial taxon of this module (Figure S7).

## 4. Discussion

Fish gut microorganisms have an essential impact on host health by participating in biological processes such as nutrient processing, detoxification, gut motility regulation, immune function, development, and mucosal tolerance [36,37]. Moreover, the study of the fish microbiome and the understanding of fish-associated microorganisms has grown significantly over the past two decades due to the emergence of nucleic-acid-based techniques for describing aquatic prokaryotes, along with the rapid growth of the aquaculture industry [38]. Indeed, research on aquaculture fish, including freshwater and marine fish farming, is very frequent due to the growing importance of aquaculture as a source of animal protein in the global food supply. In addition, aquaculture environments can be used as large-scale controlled experimental environments where fish can grow through their entire life cycle under controlled conditions, such as food, ambient temperature, water quality, etc., and thus they offer unique research opportunities that are not available in wild fish studies [39]. However, fish have unique and relatively stable interactions with a wide range of microorganisms in their environment, and studies of fish gut microbes in controlled aquaculture cannot reveal information about fish gut microbial communities in the real field. Similarly, in most cases, aquaculture environments are relatively isolated to one species of fish, which is contrary to the fact that multiple species of fish live together in the same environment under natural conditions. Further, in the process of aquaculture, in order to obtain more catches, people will control the aquaculture environment and even use drugs such as antibiotics to prevent diseases, which also affects the understanding of the real fish gut microbial community, not to mention the seasonal changes in fish gut microbial communities under real and complex natural conditions.

Chaohu Lake is the fifth largest freshwater lake in China, located in the central part of Anhui Province. It is a large natural lake in the middle and lower reaches of the Yangtze River, connected to the Yangtze River through the Yuxi River; however, more than 60 years ago, the completion of the Chaohu Lock and the Yuxi Lock made Chaohu Lake into a semi-enclosed lake with an artificially controlled water level, relatively little water exchange, and a relatively stable environment for the water bodies within the lake. Since 1 January 2019, a total fishing ban has been implemented in Chaohu Lake, which enables the fish in the lake to naturally form their respective ecological niches without the influence of human fishing and form a natural fish community in Chaohu Lake. This provided excellent experimental materials for this study, with a view for obtaining more realistic and reliable information on the gut microbial communities of different fish in Chaohu Lake. Meanwhile, due to the small amount of water exchange in Chaohu Lake, unlike rivers that are affected by upstream environments, the study of seasonal changes in the gut microbial communities of fish species in Chaohu Lake is more controllable and has fewer influencing factors. Previous findings have shown that for adult wild fish, the vast majority of their gut microbial communities are similar to those in their living aquatic environments [19], implying that even for the same fish living in different geographic environments, there are natural differences in their gut microbial communities due to differences in the aquatic microbial communities of the environments in which they live, not to mention comparing the gut microbial communities of different fish in different geographic environments. Therefore, it is of practical significance to compare the similarities and differences in the gut microbial communities of different wild fish species living in the same stable water environment.

Based on the above considerations, we collected samples of seven different fish species, *H. molitrix*, *A. nobilis*, *C. auratus*, *C. alburnus*, *C. brachygnathus*, *C. carpio*, and *C. ectenes taihuensis*, from Chaohu Lake in four consecutive seasons after the implementation of the total fishery ban in Chaohu Lake, and carried out microbial community studies on the foregut and hindgut of the gut samples of these fishes. It was found that the gut microbial community structure of these fish species in Chaohu Lake was different. These fishes in Chaohu Lake showed significantly different gut microbial community structures in different seasons, and there were also significant differences in the microbial communities of the foregut of these fishes compared with those of the hindgut of these fishes. Among these wild Chaohu Lake fishes, *H. molitrix* and *A. nobilis* are filter feeders, *C. auratus* and *C. carpio* are predators, and *C. alburnus*, *C. brachygnathus*, and *C. ectenes taihuensis* are omnivores. Furthermore, we found significant differences in the gut microbial community structure of these different dietary fishes (*p* < 0.001), which is consistent with the results of previous studies that found wild fishes with similar diets to have similar gut microbial communities [19].

In this study, the seven wild fish gut microbial communities in Chaohu Lake were predominantly composed of Firmicutes, Proteobacteria(Gamma), Proteobacteria(Alpha), Actinobacteriota, and Cyanobacteria at the taxonomic level of phylum, while *Aeromonas*, *Cetobacterium*, *Clostridium sensu stricto 1*, *Romboutsia*, and *Pseudomonas* dominated at the taxonomic level of genus, excluding unclassifiable microbial taxa, during four consecutive seasons. Previous studies have demonstrated that *Aeromonas*, *Cetobacterium*, *Clostridium sensu stricto 1*, *Romboutsia*, and *Pseudomonas* are common dominant microbial groups in the gut microbial communities of freshwater fishes [10,18,40]. There are currently 32 species of the genus *Aeromonas*, consisting of facultative anaerobic, Gram-negative, rod-shaped, and non-spore-forming bacteria, approximately 1–3 μm in length [41]. In addition, they are oxidase-positive, capable of fermenting glucose, and can tolerate concentrations of NaCl ranging from 0.3% to 5%. Most of the *Aeromonas* are opportunistic microorganisms [41]. These bacteria are naturally distributed in a variety of aquatic ecosystems and are readily isolated from fish and crustaceans [41]. *Aeromonas* produce a wide variety of virulence factors. The expressions of membrane components, toxins, enzymes, and several molecules contribute to the pathogenicity of the bacteria and act in different ways, such as tissue adhesion, immune response evasion, and host cell engagement. To disseminate virulence factors, *Aeromonas* has four secretion systems responsible for the release of these cellular products into the extracellular environment or even directly into host cells [41]. Moreover, in our results, the most abundant ASV of the genus *Aeromonas* was identified at the species level as the fish pathogen *Aeromonas salmonicida* [42]. Interestingly, some *Cetobacterium* bacteria isolated from the intestines of healthy fish can be used as probiotics to resist *Aeromonas* infections [43]. Not only that, some species of the genus *Cetobacterium*, such as the widely detected in this study *Cetobacterium somerae*, can effectively improve the intestinal conditions of fish, promote fish liver health, and enhance the host antiviral immunity [44]. Additionally, it was found that *C. somerae* was able to promote the expression of insulin in zebrafish to lower blood glucose, and *C. somerae* was also able to activate the parasympathetic nervous system through the metabolite acetic acid, which promotes the expression of insulin and the ability of glucose utilization of fish and plays an important role in regulating the health of the fish [45]. *Clostridium sensu stricto 1* is a common group in the gut microbial community of freshwater fish [10], but it is also frequently found in the gut of marine mammals [46,47,48], where it often appears as a potential pathogen [49,50]. Bacteria of the genus *Romboutsia* are Gram-positive bacteria, one of the common intestinal microorganisms, and most species of *Romboutsia* originate from the gut, although some isolated strains of *Romboutsia* have originated from diseased individuals, but recent reports have shown that this group of bacteria contains a polysaccharide-synthesizing enzyme that produces (1,3;1,4)-β-d-glucans, so the role of this group of bacteria in the human and mammalian gastrointestinal tract is less clear [51,52]. *Pseudomonas* is one of the most diverse genera, and in our study the most abundant ASV of *Pseudomonas* belonged to *Pseudomonas koreensis*, which is a common causative agent of freshwater fish [53,54].

The microbial molecular ecological network approach can reveal the interrelationships among microorganisms within a community, and these network properties are very important for the robustness and stability of microbial communities in different complex ecosystems [34,35,55,56]. The analysis of gut microbial co-occurrence networks of seven different wild fish species in Chaohu Lake showed that the gut microbial networks of different species of fish varied, with some fish having simpler gut microbial network structures, such as *H. molitrix*, *C. alburnus*, *C. ectenes taihuensis*, and *A. nobilis*, while others had relatively more complex gut microbial network structures, such as *C. auratus*, *C. carpio*, and *C. brachygnathus*. The majority of the keystone taxa in the gut microbial networks of these fish were assigned to connectors and module hubs, and some of the keystone genera responsible for microbial interactions are also dominant in the fish gut microbial community, such as *Clostridium sensu stricto 1*, *Romboutsia*, and *Pseudomonas*. This suggests that these important microbial taxa and their functional modules play an important role in maintaining the stability of the fish gut microbial community. In addition, even though the study of fish gut microbes has been intensified in recent years, there are still some fish gut microbes that cannot be classified at the genus level, suggesting that there are many unknown microorganisms in the fish gut that need to be further isolated and characterized.

## 5. Conclusions

Revealing the information of different fish gut microbial communities and the interrelationships among these microbes can help to better understand the physiology and health status of fish, for instance, the screening and utilization of some fish gut probiotics. Meanwhile, when studying the gut microbial communities of wild fish with different diets and species living in the same water in inland lakes such as Chaohu Lake, where there is little water exchange and fishing is prohibited, it is possible to observe whether the diet and the hosts have shaped the gut microbial community structure of different fish and to reveal more comprehensive and realistic information about the gut microbial communities of wild fish. In this study, we investigated the gut microbial community information of seven wild fish species from Chaohu Lake during the closed fishing term in four consecutive seasons. It was found that differences in both diet and host species caused significant differences in the gut microbial community structure of the fish. Overall, the dominant microbial groups in the gut microbial communities of these fishes in Chaohu Lake were *Aeromonas*, *Cetobacterium*, *Clostridium sensu stricto 1*, *Romboutsia*, and *Pseudomonas*. Meanwhile, the co-occurrence network analysis of the fish microbial communities revealed that the gut microbial communities of *C. auratus*, *C. carpio*, and *C. brachygnathus* formed a more complex and stable microbial network structure than those of the other four species of wild fishes. In addition, some microorganisms are not only the dominant groups in the fish gut microbial community but also the keystone taxa in the fish gut microbial network structure, such as *Clostridium sensu stricto 1*, *Romboutsia*, and *Pseudomonas*. Notably, the microbial co-occurrence network relies on sample size and is influenced by the ecological niche and environment. Future studies should collect even more gut samples to reduce interference and enhance our understanding of the relationship between the gut microbial communities. Meanwhile, isolating and sequencing the genomes of novel gut microorganisms, as well as employing third-generation metagenomic sequencing and binning technologies, will uncover their specific functions and further our knowledge of fish gut microbiology.

## Figures and Tables

**Table 1:** Detailed information on the sampling of gut samples from different fish species in Chaohu Lake during different seasons.

Species		Spring	Summer	Autumn	Winter
Silver carp (*Hypophthalmichthys molitrix*)	Foregut	L3-1F, L3-2F, L3-3F, L3-4F, L3-5F	L4-1F, L4-2F, L4-3F, L4-4F, L4-5F	LF-1, LF4, LF5, L5-1F, L5-2F	BL2-1F, BL2-2F, BL2-3F, BL2-4F, BL2-5F, BL2-6F, BL2-7F
	Hindgut	L3-1B, L3-2B, L3-3B, L3-5B	L4-1B, L4-2B, L4-3B, L4-4B, L4-5B	LB1, LB2, LB3, LB4, LB5	BL2-1B, BL2-2B, BL2-3B, BL2-4B, BL2-5B, BL2-6B, BL2-7B
Bighead carp (*Aristichthys nobilis*)	Foregut	Y3-1F, Y3-2F, Y3-3F, Y3-4F, Y3-5F	Y4-1F, Y4-2F, Y4-3F, Y4-4F, Y4-5F	YF-2, YF-3	HL2-1F, HL2-3F, HL2-4F, HL2-5F, HL2-6F, HL2-7F
	Hindgut	Y3-1B, Y3-2B, Y3-3B, Y3-4B, Y3-5B	Y4-1B, Y4-2B, Y4-3B, Y4-4B, Y4-5B	YB-1, YB-2, YB-3, YB-4, YB-5	HL2-1B, HL2-2B, HL2-3B, HL2-4B, HL2-5B, HL2-6B
Crucian carp (*Carassius auratus*)	Foregut	JY3-1F, JY3-2F, JY3-3F, JY3-4F, JY3-5F, JY3-6F	JY5-1F, JY5-2F, JY5-3F, JY4-2F, JY4-4F, JY4-5F	JF-3, JF-4	JY2-1F, JY2-2F, JY2-3F, JY2-5F, JY2-7F
	Hindgut	JY3-1B, JY3-2B, JY3-3B, JY3-4B, JY3-5B, JY3-6B	JY5-1B, JY5-2B, JY5-3B, JF4-2B, JF4-4B, JF4-5B	JB-1, JB-2, JB-3, JB-4, JB-5	JY2-1B, JY2-2B, JY2-3B, JY2-4B, JY2-5B, JY2-6B, JY2-7B
Topmouth culter (*Culter alburnus*)	Foregut	QZ3-1F, QZ3-3F, QZ3-5F	QZ4-1F, QZ4-2F, QZ4-3F, QZ4-4F, QZ4-5F	QF-3, QZ5-2F, QZ5-3F, QZ5-4F, QZ5-5F	Q2-1F, Q2-2F, Q2-3F, Q2-4F, Q2-5F, Q2-6F
	Hindgut	QZ3-2B, QZ3-3B, QZ3-5B	QZ4-1B, QZ4-2B, QZ4-3B, QZ4-4B, QZ4-5B	QB-2, QB-3, QZ5-1B, QZ5-2B, QZ5-3B, QZ5-4B	Q2-1B, Q2-2B, Q2-4B, Q2-5B, Q2-6B
*Coilia brachygnathus*	Foregut	DJ3-1F, DJ3-2F, DJ3-3F, DJ3-4F, DJ3-5F, DJ3-6F	XDJ4-1, XDJ4-2, XDJ4-3, XDJ4-4, XDJ4-5		DJ1F, DJ2F, DJ3F, DJ4F, DJ5F, DJ6F, DJ7F
	Hindgut				DJ1B, DJ2B, DJ3B, DJ4B, DJ5B, DJ6B, DJ7B
Common carp (*Cyprinus carpio*)	Foregut				LY2F, LY5F, LY6F, LY7F
	Hindgut				LY2B, LY3B, LY4B, LY5B, LY6B, LY7B
Lake anchovy (*Coilia ectenes taihuensis*)		DDJ3-1, DDJ3-2, DDJ3-3, DDJ3-4, DDJ3-5, DDJ3-7	DDJ4-1, DDJ4-2, DDJ4-3, DDJ4-4, DDJ4-5, DDJ4-6		

**Table 2:** Molecular ecological network characteristics of gut microbial communities of seven different fish species in Chaohu Lake.

Species	Node	Link	Average Clustering Coefficient (avgCC)	Average Path Distance (GD)	Modularity (M)
*H. molitrix*	73	1151	0.532	1.563	0.117
*A. nobilis*	55	743	0.565	1.500	0.078
*C. auratus*	100	200	0.268	4.116	0.535
*C. alburnus*	58	678	0.465	1.592	0.133
*C. brachygnathus*	80	185	0.127	3.103	0.438
*C. carpio*	82	142	0.039	3.488	0.494
*C. ectenes taihuensis*	74	1623	0.628	1.399	0

## Data Availability

Data are contained within the article and supplementary materials.

## Supplementary Materials

The following supporting information can be downloaded at https://www.mdpi.com/article/10.3390/microorganisms12040800/s1, Figure S1. Gut microbial co-occurrence network of *H. molitrix* and the microbial community composition of different modules; Figure S2. Gut microbial co-occurrence network of *A. nobilis* and the microbial community composition of different modules; Figure S3. Gut microbial co-occurrence network of *C. auratus* and the microbial community composition of different modules; Figure S4. Gut microbial co-occurrence network of *C. alburnus* and the microbial community composition of different modules; Figure S5. Gut microbial co-occurrence network of *C. brachygnathus* and the microbial community composition of different modules; Figure S6. Gut microbial co-occurrence network of *C. carpio* and the microbial community composition of different modules; Figure S7. Gut microbial co-occurrence network of *C. ectenes taihuensis* and the microbial community composition of module. Table S1: The original ASVs table with classified information in this study.
