# Supplementary material for: Variations and Interseasonal Changes in the Gut Microbial Communities of Seven Wild Fish Species in a Natural Lake with Limited Water Exchange during the Closed Fishing Season
Source: Microorganisms. 2024 Apr 16;12(4):800. doi: 10.3390/microorganisms12040800 (PMC11052518; doi:10.3390/microorganisms12040800)
Supplement: Supplementary file 1 [file microorganisms-12-00800-s001.zip › Supplementary_Figures.pdf]

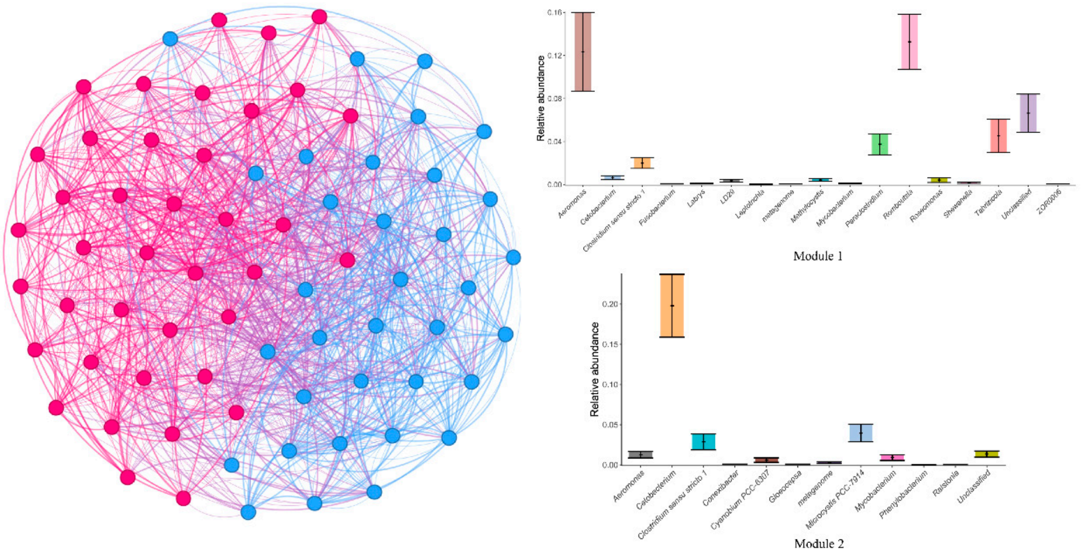

Figure S1. Gut microbial co-occurrence network of *H. molitrix*, and the microbial community composition of different modules.

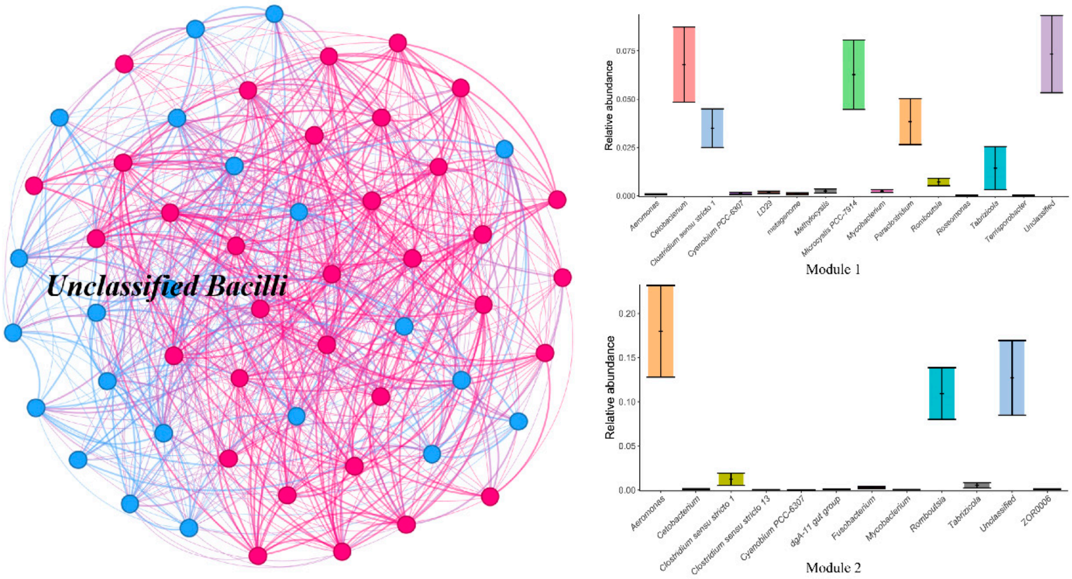

Figure S2. Gut microbial co-occurrence network of *A. nobilis*, and the microbial community composition of different modules.

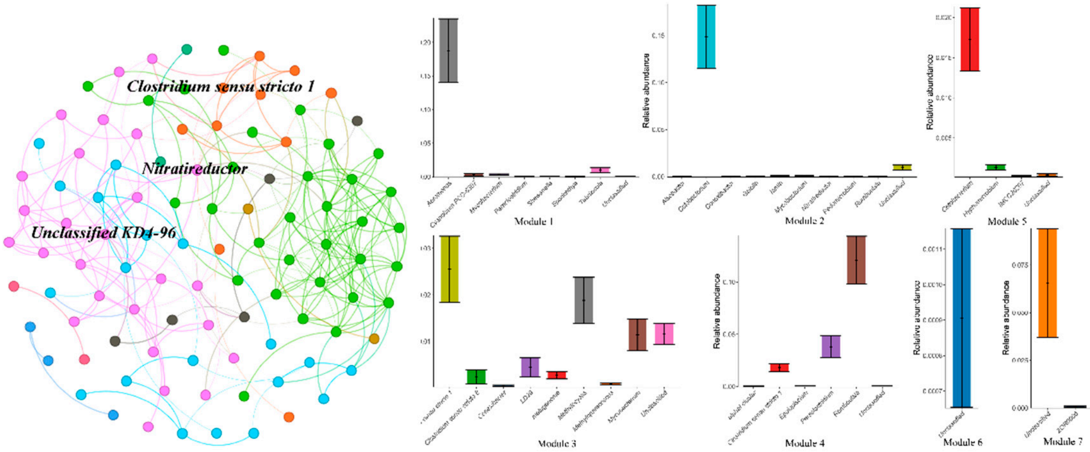

Figure S3. Gut microbial co-occurrence network of *C. auratus*, and the microbial community composition of different modules.

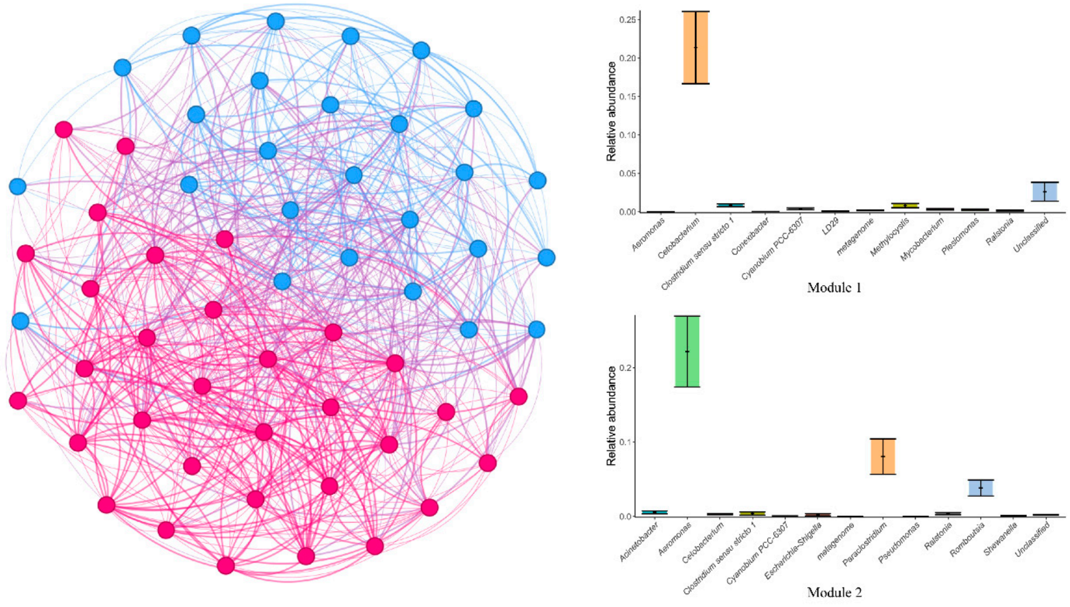

Figure S4. Gut microbial co-occurrence network of *C. alburnus*, and the microbial community composition of different modules.

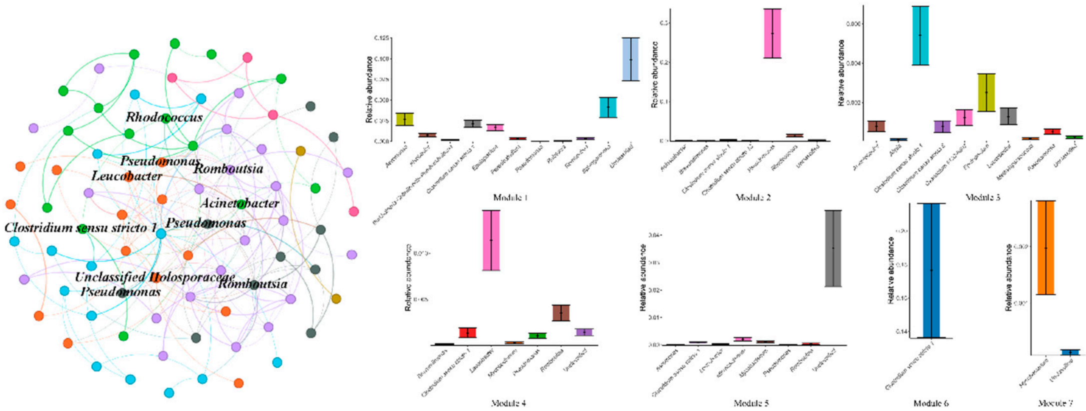

Figure S5. Gut microbial co-occurrence network of *C. brachygnathus*, and the microbial community composition of different modules.

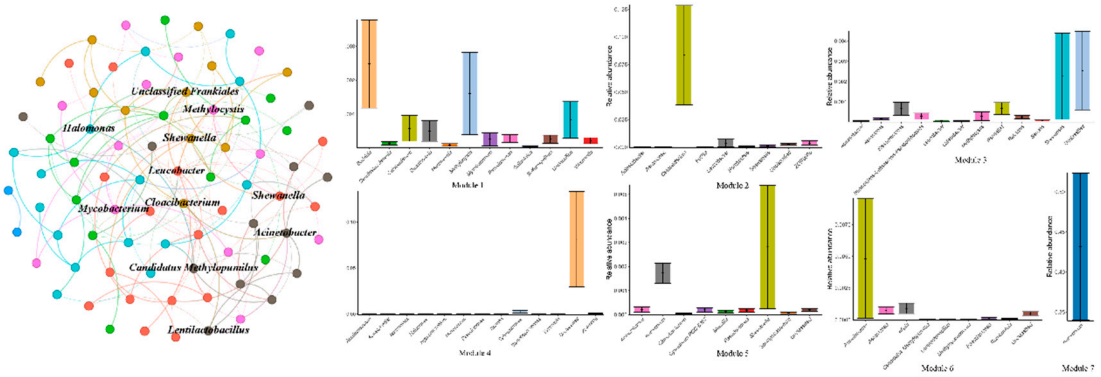

Figure S6. Gut microbial co-occurrence network of *C. carpio*, and the microbial community composition of different modules.

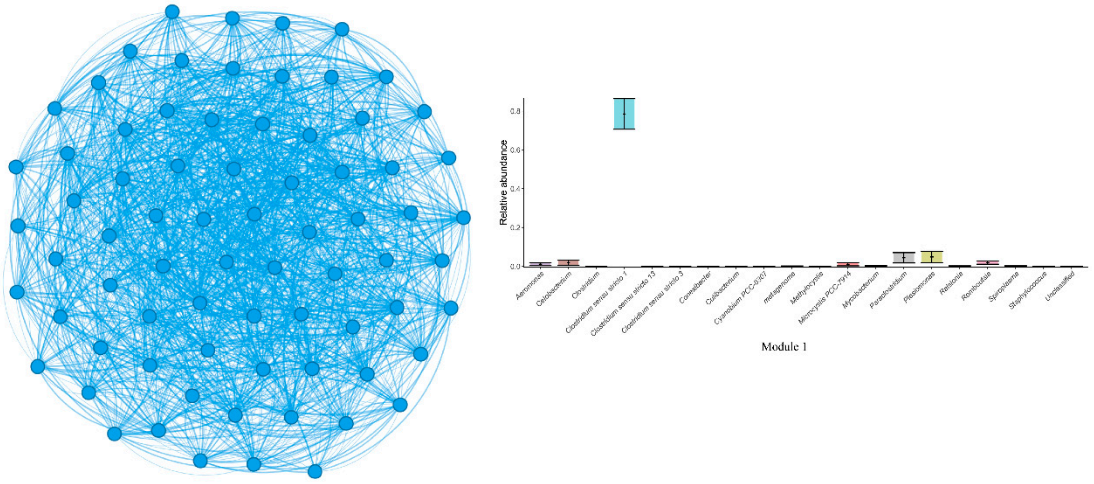

Figure S7. Gut microbial co-occurrence network of *C. ectenes taihuensis*, and the microbial community composition of module.
